# Patterns of metastasis in colon and rectal cancer

**DOI:** 10.1038/srep29765

**Published:** 2016-07-15

**Authors:** Matias Riihimäki, Akseli Hemminki, Jan Sundquist, Kari Hemminki

**Affiliations:** 1 Division of Molecular Genetic Epidemiology, German Cancer Research Centre (DKFZ), Heidelberg, Germany; 2 Center for Primary Health Care Research, Lund University, Malmö, Sweden; 3 Cancer Gene Therapy Group, Faculty of Medicine, University of Helsinki, Finland; 4 Helsinki University Hospital Comprehensive Cancer Center, Helsinki, Finland

## Abstract

Investigating epidemiology of metastatic colon and rectal cancer is challenging, because cancer registries seldom record metastatic sites. We used a population based approach to assess metastatic spread in colon and rectal cancers. 49,096 patients with colorectal cancer were identified from the nationwide Swedish Cancer Registry. Metastatic sites were identified from the National Patient Register and Cause of Death Register. Rectal cancer more frequently metastasized into thoracic organs (OR = 2.4) and the nervous system (1.5) and less frequently within the peritoneum (0.3). Mucinous and signet ring adenocarcinomas more frequently metastasized within the peritoneum compared with generic adenocarcinoma (3.8 [colon]/3.2 [rectum]), and less frequently into the liver (0.5/0.6). Lung metastases occurred frequently together with nervous system metastases, whereas peritoneal metastases were often listed with ovarian and pleural metastases. Thoracic metastases are almost as common as liver metastases in rectal cancer patients with a low stage at diagnosis. In colorectal cancer patients with solitary metastases the survival differed between 5 and 19 months depending on T or N stage. Metastatic patterns differ notably between colon and rectal cancers. This knowledge should help clinicians to identify patients in need for extra surveillance and gives insight to further studies on the mechanisms of metastasis.

Colorectal cancer (CRC) is the third most common cancer worldwide^1^. Approximately 56% of patients with CRC die from their cancer^2^. Development of metastasis is a concern for patients and clinicians alike as metastasis may be fatal, causing mass-effect and meddling with homeostasis^3^,^4^,^5^. Approximately 20% of patients with CRC already have metastases at diagnosis, and this figure has been stable over the last two decades^6^. With the help of continuous developments in CRC treatment, survival rates have improved. Metastatic disease has previously has been viewed as incurable. However, in favorable settings median survival may surpass five years in patients with solitary lung or liver metastases^7^,^8^,^9^. Detecting CRC early is vital considering the negative effect on survival conferred by already metastatic cancer^6^,^10^. Although current evidence has shown a decreased cancer specific mortality from CRC in screening groups, all-cause mortality may not be improved by screening^11^.

Recent progress in metastasis research has vastly expanded our understanding of metastasis on the cellular and molecular level^3^,^4^,^12^,^13^. Unfortunately, knowledge at the epidemiological level is lacking. Efforts to investigate metastases are hampered by the fact that cancer registries seldom include any information on metastases apart from the stage at diagnosis. In this setting, it is impossible to assess metastatic spread to specific sites. Overviews of metastatic patterns across different cancers are limited to autopsy-based studies relying on approximately one thousand deaths from metastatic cancer^14^,^15^,^16^. Although these reports are impressive and important, it may not be easy to discuss magnitude of clinically relevant metastases based on autopsies. Apart from a recent Dutch study^6^, most available reports of CRC metastases are either based on single hospital series or subnational registers^17^,^18^. An alternate approach would be to investigate the causes of death in cancer, as both hospital-based registers and cause of death registers are based on the International Classification of Diseases (ICD). In the present study, we used information from two Swedish nationwide registers, the National Patient Register and Cause of Death Register. We investigated the patterns of metastasis from colon and rectal cancer to specific sites, depending on sex, age at diagnosis, histological subtype, stage, number of metastases, and whether the primary was situated in proximal or distal colon, or in the rectum. We also estimated survival depending after diagnosis of colon and rectal cancer with distant metastasis.

## Methods

This study utilized data form several linked nationwide registers in Sweden. The Swedish Family-Cancer Database includes cancer data from the Swedish Cancer Register, encompassing cancers diagnosed in Sweden since 1958. Furthermore, death causes are included from the Cause of Death Registry, as well as vital statistics^19^. The primary sites are coded by ICD’s 7^th^ revision in the Cancer Registry. Patients with CRC were identified with codes 153 and 154.0. Then anatomical location is known through the ICD code (proximal colon [153.0/.1], distal colon [153.2/.3], and rectum [154.0]). SNOMED histological codes and is included since 1993. We identified patients with the histological subtypes signet-ring adenocarcinoma and mucinous adenocarcinoma by the SNOMED codes 8480 and 8490, whereas generic adenocarcinomas are listed under the code 8140. Cancers were staged I-IV depending on the TNM (Tumor, lymph Node, distant Metastasis) stage. T-stages “T1” and “T2” were stage I, whereas T-stages “T3” and “T4” were stage II. Irrespective of T stage, positive lymph node status (N1, N2) was stage III and occurrence of distant metastases (M1) was stage IV. TNM staging is included since 2002, and is not downgraded by neoadjuvant therapy. Analysis was therefore restricted to patients diagnosed with colon or rectal cancers in years 2002 through 2012. Follow-up on metastatic information was available until the end of 2012. Patients with multiple primary cancers were excluded.

Metastatic involvement was identified from the National Patient Register and Cause of Death Register. In the National Patient Register, metastases can either be listed as the main diagnosis or as one of up to 21 accompanying diagnoses during the hospitalization. Data from all hospitalizations in Sweden are included in the National Patient Register, which reached nationwide coverage in 1987^20^. Reporting is obligatory in both public and private healthcare facilities. Alternate sources of metastatic data were causes of death. These were identified from the national Cause of Death Registry, where the underlying causes and up to 10 accompanying causes of death are listed. Since 1996, both registers implement coding through ICD-10. The codes used for identifying metastatic sites were as follows: lung (C78.0), pleura (C78.2), other respiratory organs (C78.1/.3), peritoneum (C78.6), liver (C78.7), other gastro-intestinal (C78.4/.5/.8), urinary system (C79.0/.1), skin (C79.2), nervous system (C79.3/.4), bone (C79.5), ovary (C79.6), adrenal (C79.7), other specified (C79.8). The last-mentioned group cannot be specified further, but it includes metastases to genital organs and breast tissue. Metastases to “ill-defined” sites or unspecified sites were not included in this analysis, nor were patients with unknown anatomical site or histology. Although nodal metastases are important for staging, these were excluded due to their small number and to limit the size of this study. In certain analyses, rare sites of metastases (skin, ovary, adrenal, and other specified) were grouped together as “other metastasis”. Due to the small frequencies, these sites were analyzed together as one group.

A multivariable logistic regression model was used to calculate odds ratios (OR) for sex, age, anatomical site, and histological type on specific metastases. P-values for 2 × 2- tables were obtained by using chi-square test. Survival in metastatic colon and rectal cancers was investigated with a multivariable Cox regression model, yielding hazard ratios (HR). This model adjusted for sex, age, anatomical site, histological type, and stages T and N. All calculations were performed using SAS software, version 9.3.

## Results

**Figure 1:**
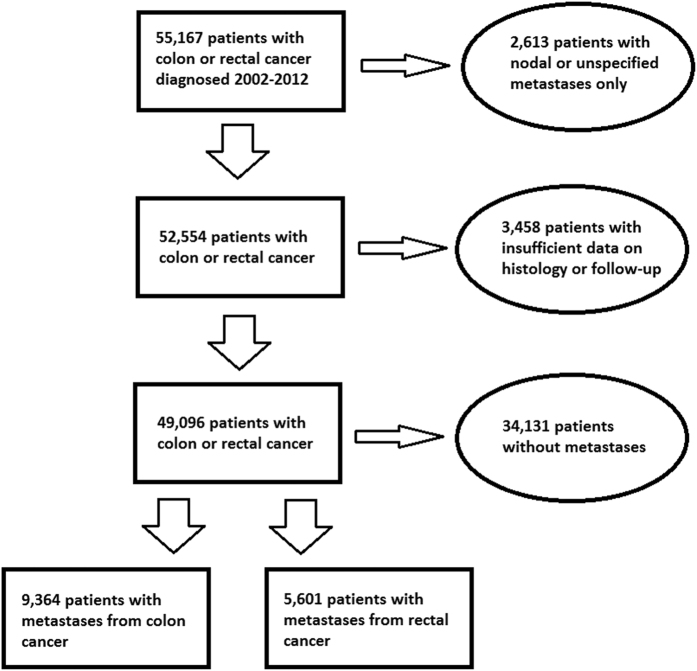
Flow chart depicting the selection of patients.

A flow chart depicts the selection of patients (Fig. 1). The number of patients, sex, histological subtype, and stage are summarized in Table 1. We identified 49,096 patients, 31,285 with colon cancer and 17,811 with rectal cancer. Of these, 9,364/5,601 (30% colon/31% rectum) had a recorded metastasis. Of them, 5,210/2,780 had a single metastasis. Metastases were obtained from the National Patient Register (8,691/5,164) and the Cause of Death Register (3,910/2,297). Mucinous and signet ring adenocarcinoma were more common in women and in patients with proximal colon cancer. The overall median age at diagnosis was 72.4 years. Patients with proximal colon cancer had the highest age at diagnosis, while rectal cancer patients had the lowest. 83% of all stage IV colorectal cancer patients diagnosed 2002 through 2012 had site specific metastases. Furthermore, 2,613 patients (5% of total) without specified extranodal metastases had metastases to lymph node, ill-defined, or unspecific sites. Of these, 442 were patients with stage IV cancer. Also, 5% had not died during the follow-up time, thus lacking the causes of death. Therefore, the underreporting in the combined registers can be assumed to be maximally only 7% for the stage IV disease, and we find no reason to believe why this underreporting would be anything else than random.

**Figure 2:**
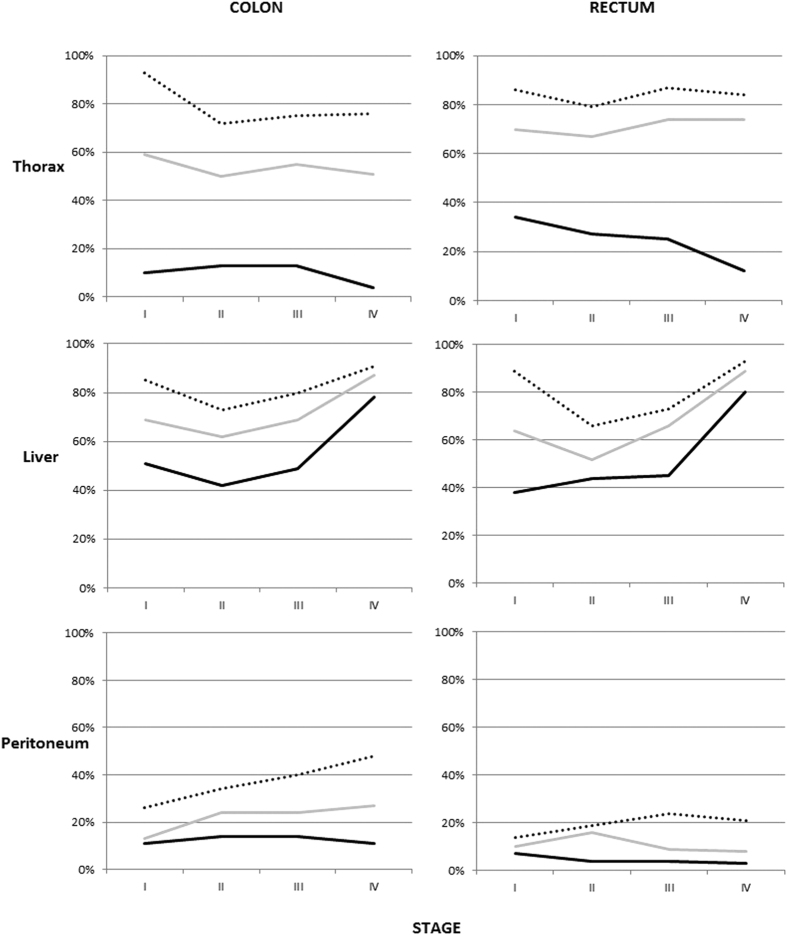
Frequency of thoracic, liver, and peritoneal metastases in patients with colon or rectal cancer, depending on how many metastases were present and the stage of patients. Black line: one metastasis, gray line: two metastases, dotted line: three or more metastases.

We described the relative frequency of specific metastases depending on the stage at diagnosis and the number of listed metastases, separately for colon or rectal cancers. The complete data can be found in Supplemental Table 1 (colon cancer) and 2 (rectal cancer). Thoracic, liver, and peritoneal metastases have been selected to be depicted in Fig. 2. In patients with single metastases, the relative frequency of thoracic metastases was higher in lower stages (p-value for difference <0.0001 in both two comparisons), especially in rectal cancer. However, there was no significant difference in the frequency of patients with thoracic metastases among those with multiple metastases. This is opposite to liver metastases, which were relative more frequent with higher stage irrespective of sex, anatomical sites, or the number of metastases (p-value < 0.0001 in all 6 comparisons). The relative frequency of peritoneal metastases was more stable across stages and numbers of metastases. Nervous system and bone metastases had a similar trend as thoracic metastases. They were uncommon as single metastases in patients with high stage (1%) but significantly more common as single metastases in patients at lower stages. As many as 10% of patients with single metastases from stage I colon cancer had bone metastasis.

Table 2 gives multivariable ORs for colon cancer patients to have metastatic spread to a single location. Women had a lower OR to develop liver (OR= 0.80 [95% CI: 0.73–0.91]) and bone metastases (0.36) compared to men. For most sites, younger patients had a higher risk. Mucinous and signet ring adenocarcinomas had a lower risk of liver metastases (0.48 [0.41–0.57]) but a higher risk of peritoneal metastases (3.80 [3.18–4.55]). Distal colon cancer was associated with an increased risk of thoracic metastases.

Compared with colon cancer, rectal tumors (Table 3) spread significantly more frequently to the thorax (2.41 [2.12–2.75]), the bone (1.38 [1.08–1.77]), and the nervous system (1.49 [1.09–2.04]), but less likely in the peritoneum (0.33 [0.27–0.40]). As in colon cancer, women with rectal cancer had a fewer metastases to the liver (0.77 [0.70–0.86]). The differences between age and histological type were similar as in colon cancer: increased age was associated with less peritoneal and liver metastases, although no difference in thoracic metastases could be detected.

In Supplemental Tables 3 and 4, the spread of metastases from colon and rectal cancers is shown, irrespective of their number of metastases. As more metastases are included in the analysis, the differences between patient characteristics become less pronounced. Of all patients with metastatic cancer, the most common sites of metastasis were the liver (70% in colon cancer/70% in rectal cancer) and the thorax (32%/47%). In colon cancer, the third most common site was the peritoneum (21%) whereas in rectal cancer it was the bone (12%). Nervous system metastases were present in 5% of colon cancer, and in 8% of rectal cancer.

Table 4 addresses patients with multiple metastases. Liver metastases were most frequently solitary metastases (in 48% of colon/45% of rectal cancer), whereas the percentages were substantially lower for other metastatic locations. Lung metastases often occurred together with liver metastases (73%/63%). Liver metastases were less frequent among patients with peritoneal (48%/52%) or nervous system metastases (53%/46%). In contrast, lung metastases were often present in patients with nervous system metastases (54%/65%).

Survival in metastatic CRC is depicted in Table 5 for patients with a single metastatic site. The HRs were worst for old patients (2.40 [2.12–2.72] for those diagnosed at age >79 years vs. 1.00 for age <60 years), for metastatic locations in the nervous system (1.73 [1.21–2.49]) or the bone (1.56 [1.13–2.16]) compared the liver (1.00) or the thorax (0.64 [0.55–0.76]), and for proximal colon (1.42 [1.29–1.56] vs. 1.00 for the rectum). Median survival for patients with liver metastases (9 months) was shorter than for those with thoracic metastases (14 months); the survival was worst for rare patients with sole nervous system (4 months) or bone (5.5 months) metastases. The T and N stages strongly influenced survival. Patients with T2 survived 16.5 months compared to those with T4 (8 months) or TX (5 months). Patients with N0 survived 19 months compared to those with N2 (8 months) or NX (5 months). Results did not differ if all stage IV patients were considered, irrespective of the number of metastases (data not shown).

### Ethical Approval

This study was approved by the ethical committee at Lund University, Sweden.

## Discussion

In this paper, we investigated metastatic patterns in colon and rectal cancers and presented results from nationwide Swedish registers. Significant variations could be noted between anatomical sites of primary cancer and its histological subtypes. These patterns have not been described earlier to this extent and address questions regarding the nature of metastatic spread and biology of metastases. Although the prognosis is indeed already dire in metastatic colon and rectal cancers, the site of distant metastasis was an important prognostic factor. Also, T- and N-stage had a huge impact on survival, and depending on these factors the median survival could range between 5 and 19 months. Therefore, stage IV cancer should not be considered as a single entity. Overall, median survival after diagnosis of colon or rectal cancer was 10 months, which is in line with previous observations^6^.

The “anatomical/mechanical” and “seed-and-soil” hypotheses are widely accepted to explain metastatic spread^3^,^5^,^21^,^22^. Recent developments have refined the seed-and-soil hypothesis to new levels, because it has been possible to investigate tumor-stroma interactions at a molecular level. Specific tumor cells may show a preference to specific target organs of metastasis^23^,^24^,^25^. For example, small cell lung cancer is more prone to metastasize to the liver than other histological types^5^. Previous autopsy based studies have put forward theories about the cascadic spread of gastro-intestinal tumors^21^,^22^. Metastases at the first draining site may act as seeds to further metastasis. From the colon and proximal parts of the rectum, blood is drained through the portal system to the liver. From the liver the next organ is the lungs, via the heart. The distal parts of the rectum surpass the liver, and the first encountered organ is the lungs. Therefore it seems logical that we observed that rectal cancer more frequently metastasized to thoracic organs than colon cancer, which is in line with previous suggestions^6^,^18^. Also, all sites in the gastro-intestinal system share a common lymphatic drain, flowing via the cisterna chyli to the left subclavian vein, and to the lungs. Adding to the complexity, metastases may spread through the peritoneal fluid within the peritoneal cavity. Mucinous adenocarcinomas showed excessive metastasizing within the peritoneum. The production of mucus may enable access to the peritoneal space^26^. Mucinous adenocarcinomas are thought of to be more aggressive. This may enhance growth in the peritoneal lining as soon as the peritoneum is accessed, although it is also possible that mucinous adenocarcinomas may have a genetic advantage to growth in the peritoneal cavity. Although the differences between colon and rectal cancer were striking, there were also notable differences between the proximal and distal colon, clearly demonstrating their different biology. The proximal colon originates form the midgut, whereas the distal colon stems from the hindgut. The two entities vary in e.g. epidemiology, biology, and genetics^27^.

Our findings regarding multiple metastases are important to understand the spread of metastases. Respiratory metastases were indeed more common in patients who also had metastases into bone or the nervous system, whereas respiratory metastases were infrequent together with peritoneal metastasis and other gastro-intestinal sites. Most patients with respiratory metastases also had liver metastases. Some distinct patterns can be recognized. It appears that CRC spreads via the portal circulation to the liver, and from there to the lungs. It may also reach the lungs directly, perhaps using lymphatics, or directly from the distal rectum. However, in the present data there were clear indications that the lungs seem to be an important waypoint toward further spread: nervous system metastases occurred more frequently together with respiratory metastases than with liver metastases. One speculation is that tumors metastasizing through the “normal” portal route are less mobile than more adventurous cells able to seek the lungs or the central nervous system. Further studies are clearly needed, but perhaps the latter class of cells represents classic cancer stem cells better than the more passive tumor initiating cells which just end up in the liver with the blood flow. CRC may metastasize within the abdominal cavity, giving rise to non-hematogenous ovarian metastases, which were frequently detected together with peritoneal metastases, especially in colon cancer. This is logical as the peritoneum and ovaries are often considered a continuum especially in the context of primary cancer of these organs. Furthermore, ovarian metastases were substantially more common among women with colon than rectal cancer, which may be due to anatomic factors as the intestine leaves the peritoneal double bag about midway through the rectum.

Recent research on the immunology of cancer has provided intriguing insight into the metastatic process, and may help us further understand the difference between thoracic and liver metastases. In the present study, many CRC patients presented with extrahepatic metastases and without detectable liver metastases. As discussed above, anatomical factors may be an explanation. However, one could hypothesize that liver metastases have indeed been present, but have been eliminated or entered a dormant phase, rendering treatment ineffective^12^. At lower stages, thoracic metastases were almost as frequent as liver metastases, and also in stage IV patients with multiple metastases. The only group where liver metastases were clearly more common was in stage IV patients with single metastases. Similar findings were reported in a recent Japanese hospital study^28^, where lung metastases were more frequent in CRC patients who underwent curative surgery, compared with stage IV patients. Therefore, it seems that lung metastases need a longer time to grow, compared with liver metastases. This is not an unaccustomed thought, because the liver is considered an immunosuppressive organ^29^ and survival in liver metastases was indeed poor, only 9 months. If the metastasis succeeds in escaping the immune system, the milieu of the liver will promote the growth of liver metastases, which thus will become clinically apparent. Metastases to other sites have yet not occurred, at least at a clinically detectable rate. The relatively better prognosis in thoracic metastases implies a slower growth rate, which is compatible with retained activity of anti-tumor immune responses. Improvements in radiological methods have enabled detection of event smaller tumor growths, e.g. lung metastases^6^,^18^.

In many patients, spread to the lung coincides with further metastases to the nervous system or bone, with many clinical implications. For example, the brain is not normally imaged in routine evaluation of CRC patients, regardless of metastasis location. Diagnosis of brain and bone metastases is not for intellectual interest only; it has direct links to treatment selection. Brain metastases can be treated with whole brain or stereotactic irradiation; the latter is more attractive if the metastases are found early^30^. For prevention of bone metastasis associated fractures, bisphosphonates are available^31^. Patients with sole liver metastases dominating could represent a tumor immunologically distinct from those cases where tumor load is smaller and lung and bone metastases are present. These findings could have immediate clinical implications also with regard to the recent breakthrough of checkpoint inhibiting antibodies^32^. If the subsets proposed here are immunologically distinct, they may respond to checkpoint inhibitors. There are much recent data indicating that “the Immunoscore” is quite relevant in CRC^33^. Our findings provide further hints that not all colorectal tumors are alike immunologically or metastatically.

An unfortunate shortcoming in most population based analyses, including ours, is the lack of diagnostic and other clinical data. This includes for example imaging, comorbidities, or molecular profiling. Also, we cannot consider the effect of treatment received. Our inclusion of death certificates is an important strength over previous reports, because we can thereby access those cancer patients who died from indeed clinically relevant metastases but were not hospitalized for unknown reasons.

In summary, we provide reliable figures on the metastatic spread from colon and rectal cancer. Anatomical location and histological subtype profoundly affected the patterns of metastasis when considering organs beyond the liver: rectal cancer metastasized to the thorax, nervous system and bone, whereas colon cancer and mucinous and signet ring adenocarcinomas metastasized within the peritoneum. Several distinct combinations of metastatic occurred with likely clues to the metastatic pathways. Prognosis in CRC patients with solitary metastases was worst in nervous system (4 months) and bone (5.5 months) metastases, intermediary in liver metastases (9 months) and most favorable with thoracic metastases (14 months). The T and N stages were important predictors of survival in CRC patients with solitary metastases. Careful surveillance of lung metastases in lower stage patients is warranted, most importantly in rectal cancer.

## Additional Information

**How to cite this article**: Riihimäki, M. *et al*. Patterns of metastasis in colon and rectal cancer. *Sci. Rep.* **6**, 29765; doi: 10.1038/srep29765 (2016).

## Supplementary Material

Supplementary Information

## Figures and Tables

**Table 1:** Distribution of stage, histological type, and median age at diagnosis (years) by anatomical site, and sex in 49,096 patients with colon or rectal cancer.

	Men			Women		
	N	%	Age	N	%	Age
**Proximal colon**						
All	7969	31%	73	9754	42%	76
Stage						
*I*	867	11%	74	1034	11%	77
*II*	2451	31%	75	3050	31%	77
*III*	2076	26%	73	2691	28%	76
*IV*	1366	17%	70	1533	16%	73
*Unknown*	1209	15%	74	1446	15%	77
Histology						
AD*	7123	89%	73	8543	88%	76
SR/MC**	846	11%	73	1211	12%	77
**Distal colon**						
All	7367	29%	71	6195	27%	72
Stage						
*I*	1069	15%	72	968	16%	73
*II*	1908	26%	73	1571	25%	74
*III*	1769	24%	70	1456	24%	70
*IV*	1358	18%	69	1076	17%	68
*Unknown*	1263	17%	73	1124	18%	74
Histology						
*AD*	6911	94%	71	5733	93%	72
*SR/MC*	456	6%	69	462	7%	72
**Rectum**						
All	10477	41%	70	7334	31%	72
Stage						
*I*	1941	19%	70	1484	20%	71
*II*	2085	20%	70	1339	18%	72
*III*	2262	22%	69	1662	23%	70
*IV*	1720	16%	68	1078	15%	70
*Unknown*	2469	24%	72	1771	24%	75
Histology						
*AD*	10036	96%	70	7031	96%	72
*SR/MC*	441	4%	69	303	4%	72
Total (Row %)	25813	100%	71	23283	100%	74

*AD = Adenocarcinoma. **SR/MC = signet ring/mucinous adenocarcinoma.

**Table 2:** Multivariable logistic regression model for odds for specific metastases in colon cancer patients with a single metastasis.

Patient characteristics	Any metastasis			Thorax				Peritoneum				Liver				Other Gastro-intestinal				Nervous system				Bone				Other			
	OR	95% CI		%	OR	95% CI		%	OR	95% CI		%	OR	95% CI		%	OR	95% CI		%	OR	95% CI		%	OR	95% CI		%	OR	95% CI	
All				8%				4%				62%				5%				2%				3%				8%			
Sex																															
*Men*	1			8%	1			12%	1			64%	1			4%	1			2%	1			4%	1			7%	1		
*Women*	0.9	0.8	0.9	8%	0.9	0.7	1.1	14%	1.1	0.9	1.3	59%	0.8	0.7	0.9	5%	1.2	0.9	1.5	2%	1.1	0.7	1.7	2%	0.4	0.3	0.5	10%	**1.3**	1.1	1.6
Age at diagnosis																															
<*60*	1			7%	1			17%	1			62%	1			4%	1			1%	1			3%	1			4%	1		
*60–69*	0.9	0.8	1.0	7%	1.0	0.7	1.4	13%	0.7	0.6	0.9	64%	0.9	0.8	1.1	4%	1.0	0.7	1.6	1%	1.5	0.6	3.3	3%	1.0	0.6	1.7	5%	1.1	0.8	1.6
*70–79*	0.8	0.7	0.8	10%	1.2	0.9	1.6	11%	0.5	0.4	0.6	62%	0.8	0.7	0.9	5%	1.0	0.7	1.5	2%	1.5	0.7	3.4	3%	0.9	0.6	1.5	5%	1.0	0.7	1.3
>*79*	0.6	0.6	0.7	10%	1.1	0.8	1.5	11%	0.4	0.3	0.6	59%	0.6	0.6	0.7	4%	0.7	0.5	1.1	2%	1.6	0.7	3.6	3%	0.9	0.5	1.6	10%	1.0	0.7	1.4
Anatomical site																															
*Proximal colon*	1			7%	1			13%	1			61%	1			5%	1			2%	1			3%	1			8%	1		
*Distal colon*	**1.1**	1.0	1.2	10%	**1.4**	1.2	1.7	13%	1.1	0.9	1.3	62%	1.1	1.0	1.1	4%	0.7	0.5	0.9	1%	0.6	0.4	0.9	3%	1.1	0.8	1.5	9%	**1.2**	1.0	1.5
Histology																															
*Adenocarcinoma*	1			8%	1			10%	1			64%	1			4%	1			2%	1			3%	1			8%	1		
*Signet ring/Mucinous AD*	0.9	0.8	1.0	8%	0.9	0.6	1.3	39%	**3.8**	3.2	4.5	35%	0.5	0.4	0.6	5%	0.7	0.4	1.2	2%	0.7	0.3	1.6	3%	1.1	0.6	1.9	11%	1.3	1.0	1.8

The model adjusts for sex, age at diagnosis, anatomical site, and histological type.

Bold values indicate significantly higher odds, and underlined values indicate significantly lower odds.

**Table 3:** Multivariable logistic regression model for odds of specific metastases in rectal cancer patients with a single metastasis.

Patient characteristics	Any metastasis			Thorax				Peritoneum				Liver				Other Gastro-intestinal				Nervous system				Bone				Other			
	OR	95% CI		%	OR	95% CI		%	OR	95% CI		%	OR	95% CI		%	OR	95% CI		%	OR	95% CI		%	OR	95% CI		%	OR	95% CI	
All (OR rectum vs. colon)	1.0	0.9	1.0	19%	**2.4**	2.1	2.7	4%	0.3	0.3	0.4	61%	0.9	0.9	1.0	2%	0.4	0.3	0.6	3%	**1.5**	1.1	2.0	4%	**1.4**	1.1	1.8	7%	0.9	0.8	1.1
Sex																															
*Men*	1			18%	1			4%	1			65%	1			2%	1			3%	1			4%	1			5%	1		
*Women*	0.9	0.8	1.0	22%	1.2	1.0	1.4	4%	1.1	0.8	1.6	55%	0.8	0.7	0.9	2%	0.7	0.4	1.1	3%	0.9	0.6	1.4	3%	0.7	0.5	1.0	11%	**1.9**	1.4	2.5
Age at diagnosis																															
<*60*	1			15%	1			5%	1			65%	1			1%	1			3%	1			7%	1			16%	1		
*60–69*	0.9	0.8	1.0	18%	1.1	0.9	1.5	4%	0.7	0.4	1.1	66%	0.9	0.8	1.1	1%	0.4	0.2	1.2	4%	0.7	0.4	1.5	6%	0.9	0.5	1.6	15%	0.7	0.5	1.1
*70–79*	0.8	0.7	0.9	22%	1.2	0.9	1.6	4%	0.8	0.5	1.3	57%	0.7	0.6	0.8	3%	1.4	0.7	3.1	4%	1.2	0.6	2.2	6%	1.1	0.6	1.8	14%	0.7	0.5	1.1
>*79*	0.7	0.6	0.8	22%	1.1	0.8	1.4	3%	0.5	0.3	0.9	54%	0.6	0.5	0.7	3%	1.8	0.8	3.9	5%	0.7	0.3	1.4	11%	1.1	0.6	1.9	16%	1.0	0.7	1.5
Histology																															
*Adenocarcinoma*	1			19%	1			4%	1			62%	1			2%	1			3%	1			4%	1			7%	1		
*Signet ring/Mucinous AD*	1.0	0.8	1.2	20%	1.0	0.7	1.5	11%	**3.2**	1.8	5.6	42%	0.6	0.5	0.9	2%	1.3	0.4	4.1	1%	0.3	0.0	2.2	7%	1.9	1.0	3.8	16%	**2.5**	1.6	4.0

The model adjusts for sex, age at diagnosis, and histological type.

Bold values indicate significantly higher odds, and underlined values indicate significantly lower odds.

**Table 4:** Location of metastases in patients with spread to one (N = 7,990) or more (N = 6,975) sites.

Site of metastasis	Total amount	As one of N listed metastases				% multiple	Lung	Pleura/Mediastinum	Peritoneum	Liver	Other G-I	Urinary system	Skin	Nervous system	Bone	Adrenal	Ovary*	Other
		1	2	3	4+													
**COLON**																		
Lung	2881	410	1406	718	347	86%	100%	5%	15%	73%	7%	1%	1%	8%	11%	3%	2%	11%
Pleura/Med.	260	16	75	82	87	94%	54%	100%	30%	60%	11%	2%	1%	10%	12%	2%	2%	18%
Peritoneum	2000	652	728	395	225	67%	21%	4%	100%	48%	11%	2%	3%	2%	4%	1%	5%	14%
Liver	6587	3129	2200	873	385	52%	32%	2%	14%	100%	7%	1%	1%	3%	6%	1%	1%	8%
Other G-I	909	230	317	221	141	75%	23%	3%	24%	50%	100%	5%	3%	2%	5%	1%	3%	16%
Urinary system	217	72	67	42	36	67%	19%	2%	18%	32%	19%	100%	3%	4%	5%	4%	1%	19%
Skin	168	40	47	38	43	76%	25%	2%	30%	42%	15%	4%	100%	5%	11%	1%	2%	23%
Nervous system	429	90	104	134	99	79%	54%	6%	7%	53%	5%	2%	2%	100%	17%	6%	1%	10%
Bone	717	145	203	203	166	80%	45%	4%	10%	59%	7%	1%	3%	10%	100%	4%	2%	16%
Adrenal	139	8	43	43	45	94%	55%	4%	12%	65%	6%	6%	1%	17%	19%	100%	1%	14%
Ovary	205	45	58	57	45	78%	22%	2%	47%	43%	14%	1%	2%	2%	7%	1%	100%	18%
Other	1126	373	250	288	215	67%	29%	4%	25%	50%	13%	4%	3%	4%	10%	2%	3%	100%
**RECTUM**																		
Lung	2591	550	1267	547	227	79%	100%	4%	6%	63%	6%	2%	1%	11%	14%	3%	0%	9%
Pleura/Med.	156	11	43	55	47	93%	74%	100%	10%	58%	4%	4%	3%	13%	18%	3%	0%	15%
Peritoneum	461	115	155	124	67	75%	32%	3%	100%	52%	13%	3%	3%	2%	11%	2%	1%	15%
Liver	3898	1769	1354	556	219	55%	42%	2%	6%	100%	6%	1%	1%	5%	10%	2%	0%	7%
Other G-I	377	57	141	119	60	85%	38%	2%	15%	58%	100%	5%	2%	5%	13%	3%	1%	12%
Urinary system	115	13	28	45	29	89%	42%	5%	13%	43%	16%	100%	3%	11%	19%	8%	2%	31%
Skin	83	16	20	26	21	81%	46%	5%	16%	40%	10%	5%	100%	11%	22%	7%	1%	18%
Nervous system	442	75	129	142	98	83%	65%	5%	2%	46%	4%	3%	2%	100%	18%	7%	0%	13%
Bone	681	118	194	227	142	83%	54%	4%	7%	57%	7%	3%	3%	11%	100%	5%	0%	13%
Adrenal	108	8	19	28	53	93%	70%	5%	8%	58%	9%	8%	6%	30%	31%	100%	0%	15%
Ovary	34	11	8	11	4	68%	26%	0%	15%	41%	6%	6%	3%	3%	6%	0%	100%	24%
Other	602	37	293	168	104	94%	40%	4%	11%	43%	8%	6%	2%	9%	14%	3%	1%	100%

Percentages indicate how many of all patients with site-specific metastases have multiple metastases also involving other sites.

**Table 5:** Median survival (months) and multivariable HR for death after diagnosis of metastatic (M1) colorectal cancer, by sex, primary site, histological subtype, age at diagnosis, stage, and metastatic site.

Characteristic	N	HR	95% CI		Median survival (months)
Total	3379				9
Sex					
*Men*	1902	**1**	1	1	10
*Women*	1477	1.01	0.93	1.09	8
Age at diagnosis					
*<60*	673	1	1	1	12
*60–69*	1011	1.09	0.97	1.23	11
*70–79*	1007	**1.60**	1.43	1.80	8
*>79*	688	**2.40**	2.12	2.72	5
T stage					
*T1*	35	1.38	0.91	2.11	10
*T2*	90	0.63	0.47	0.85	16.5
*T3*	1331	1	1	1	13
*T4*	933	**1.53**	1.39	1.70	8
*TX*	930	**2.07**	1.77	2.42	5
N stage					
*N0*	476	1	1	1	19
*N1*	710	**1.52**	1.31	1.77	13
*N2*	1113	**2.09**	1.82	2.40	8
*NX*	1080	**2.23**	1.87	2.65	5
Metastatic site					
*Thorax*	220	0.64	0.55	0.76	14
*Peritoneum*	271	0.81	0.70	0.95	7
*Liver*	2643	1	1	1	9
*Gastro-intestinal*	48	0.72	0.53	0.99	6.5
*Bone*	42	**1.56**	1.13	2.16	5.5
*Nervous system*	32	**1.73**	1.21	2.49	4
*Other*	123	0.87	0.71	1.07	9
Anatomical site					
*Proximal colon*	1051	**1.42**	1.29	1.56	6
*Distal colon*	1202	0.96	0.87	1.07	11
*Rectum*	1126	1	1	1	11
Histological subtype					
*Adenocarcinoma*	3153	1	1	1	9
*Signet ring/Mucinous*	226	0.83	0.70	0.98	10

Only patients with one distant metastasis are included.

Bold values indicate significantly higher hazard, and underlined values indicate significantly lower hazard.
