# Supplementary material for: Patterns of metastasis in colon and rectal cancer
Source: Sci Rep. 2016 Jul 15;6:29765. doi: 10.1038/srep29765 (PMC4945942; doi:10.1038/srep29765)
Supplement: Supplementary Information [file srep29765-s1.doc]

PATTERNS OF METASTASIS IN COLON AND RECTAL CANCER

Supplementary material

Matias Riihimäki, M.D. Ph.D.^1,2^, Akseli Hemminki, M.D. Ph.D.^3,4^, Jan Sundquist, Prof. M.D.^2^,and Kari Hemminki, Prof. M.D.^1,2^

^1^Division of Molecular Genetic Epidemiology, German Cancer Research Centre (DKFZ), Heidelberg, Germany.

^2^Center for Primary Health Care Research, Lund University, Malmö, Sweden.

^3^ Cancer Gene Therapy Group, Faculty of Medicine, University of Helsinki, Finland

^4^ Helsinki University Hospital Comprehensive Cancer Center, Helsinki, Finland.

Corresponding Author:

Matias Riihimäki

Division of Molecular Genetic Epidemiology, German Cancer Research Centre (DKFZ), Heidelberg, Germany

E-mail: matias.riihimaki@gmail.com

Word count: 198 (abstract), 3,246 (text), 5 tables, 2 figures, 4 supplemental tables

Running title: Metastasis in colon and rectal cancer

| **Supplemental Table 1.** Distribution of metastases in colon cancer by stage and number of metastases. P-values are two-sided, comparing the proportion of specific metastases between stages, separately depending on the number of metastases. | | | | | | | | | | | | | | | | | | | | | | | | |
| --- | --- | --- | --- | --- | --- | --- | --- | --- | --- | --- | --- | --- | --- | --- | --- | --- | --- | --- | --- | --- | --- | --- | --- | --- |
| **Stage** | **Number of metastases** | **Patients with metastases** | | **Thoracic** | | | **Peritoneum** | | | **Liver** | | | **Other gastro-intestinal** | | | **Nervous system** | | | **Bone** | | | **Other** | | |
|  |  | **N** | **%** | **N** | **%** | **p** | **N** | **%** | **p** | **N** | **%** | **p** | **N** | **%** | **p** | **N** | **%** | **p** | **N** | **%** | **p** | **N** | **%** | **p** |
| I | 1 | 134 | 100% | 13 | 10% |  | 15 | 11% |  | 69 | 51% |  | 6 | 4% |  | 5 | 4% |  | 14 | 10% |  | 12 | 9% |  |
|  | 2 | 61 | 100% | 36 | 59% |  | 8 | 13% |  | 42 | 69% |  | 6 | 10% |  | 7 | 11% |  | 10 | 16% |  | 11 | 18% |  |
|  | 3+ | 27 | 100% | 25 | 93% |  | 7 | 26% |  | 23 | 85% |  | 2 | 7% |  | 4 | 15% |  | 9 | 33% |  | 15 | 56% |  |
|  |  |  |  |  |  |  |  |  |  |  |  |  |  |  |  |  |  |  |  |  |  |  |  |  |
| II | 1 | 662 | 100% | 89 | 13% |  | 90 | 14% |  | 275 | 42% |  | 52 | 8% |  | 17 | 3% |  | 34 | 5% |  | 104 | 16% |  |
|  | 2 | 276 | 100% | 138 | 50% |  | 66 | 24% |  | 170 | 62% |  | 44 | 16% |  | 17 | 6% |  | 23 | 8% |  | 76 | 28% |  |
|  | 3+ | 134 | 100% | 97 | 72% |  | 45 | 34% |  | 98 | 73% |  | 53 | 40% |  | 22 | 16% |  | 34 | 25% |  | 80 | 60% |  |
|  |  |  |  |  |  |  |  |  |  |  |  |  |  |  |  |  |  |  |  |  |  |  |  |  |
| III | 1 | 1163 | 100% | 147 | 13% |  | 164 | 14% |  | 568 | 49% |  | 73 | 6% |  | 26 | 2% |  | 38 | 3% |  | 147 | 13% |  |
|  | 2 | 637 | 100% | 353 | 55% |  | 156 | 24% |  | 442 | 69% |  | 88 | 14% |  | 24 | 4% |  | 51 | 8% |  | 139 | 22% |  |
|  | 3+ | 364 | 100% | 273 | 75% |  | 144 | 40% |  | 293 | 80% |  | 102 | 28% |  | 66 | 18% |  | 79 | 22% |  | 210 | 58% |  |
|  |  |  |  |  |  |  |  |  |  |  |  |  |  |  |  |  |  |  |  |  |  |  |  |  |
| IV | 1 | 2254 | 100% | 93 | 4% | <0.0001 | 241 | 11% | 0.002 | 1747 | 78% | <0.0001 | 42 | 2% | <0.0001 | 21 | 1% | 0.001 | 23 | 1% | <0.0001 | 87 | 4% | <0.0001 |
|  | 2 | 1410 | 100% | 724 | 51% | 0.2 | 383 | 27% | 0.006 | 1222 | 87% | <0.0001 | 116 | 8% | <0.0001 | 40 | 3% | 0.0004 | 81 | 6% | 0.04 | 243 | 17% | 0.0004 |
|  | 3+ | 741 | 100% | 561 | 76% | 0.17 | 357 | 48% | 0.001 | 677 | 91% | <0.0001 | 151 | 20% | <0.0001 | 97 | 13% | 0.17 | 192 | 26% | 0.33 | 344 | 46% | 0.0007 |
|  |  |  |  |  |  |  |  |  |  |  |  |  |  |  |  |  |  |  |  |  |  |  |  |  |

| **Supplemental Table 2.** Distribution of metastases in rectal cancer by stage and number of metastases. P-values are two-sided, comparing the proportion of specific metastases between stages, separately depending on the number of metastases. | | | | | | | | | | | | | | | | | | | | | | | | |
| --- | --- | --- | --- | --- | --- | --- | --- | --- | --- | --- | --- | --- | --- | --- | --- | --- | --- | --- | --- | --- | --- | --- | --- | --- |
| **Stage** | **Number of metastases** | **Patients with metastases** | | **Thoracic** | | | **Peritoneum** | | | **Liver** | | | **Other gastro-intestinal** | | | **Nervous system** | | | **Bone** | | | **Other** | | |
|  |  | **N** | **%** | **N** | **%** | **p** | **N** | **%** | **p** | **N** | **%** | **p** | **N** | **%** | **p** | **N** | **%** | **p** | **N** | **%** | **p** | **N** | **%** | **p** |
| I | 1 | 151 | 100% | 51 | 34% |  | 11 | 7% |  | 57 | 38% |  | 5 | 3% |  | 4 | 3% |  | 6 | 4% |  | 17 | 11% |  |
|  | 2 | 70 | 100% | 49 | 70% |  | 7 | 10% |  | 45 | 64% |  | 9 | 13% |  | 8 | 11% |  | 6 | 9% |  | 12 | 17% |  |
|  | 3+ | 37 | 100% | 32 | 86% |  | 5 | 14% |  | 33 | 89% |  | 8 | 22% |  | 11 | 30% |  | 15 | 41% |  | 14 | 38% |  |
|  |  |  |  |  |  |  |  |  |  |  |  |  |  |  |  |  |  |  |  |  |  |  |  |  |
| II | 1 | 342 | 100% | 91 | 27% |  | 13 | 4% |  | 150 | 44% |  | 10 | 3% |  | 16 | 5% |  | 16 | 5% |  | 46 | 13% |  |
|  | 2 | 150 | 100% | 101 | 67% |  | 24 | 16% |  | 78 | 52% |  | 11 | 7% |  | 16 | 11% |  | 24 | 16% |  | 38 | 25% |  |
|  | 3+ | 89 | 100% | 70 | 79% |  | 17 | 19% |  | 59 | 66% |  | 25 | 28% |  | 24 | 27% |  | 29 | 33% |  | 54 | 61% |  |
|  |  |  |  |  |  |  |  |  |  |  |  |  |  |  |  |  |  |  |  |  |  |  |  |  |
| III | 1 | 578 | 100% | 146 | 25% |  | 22 | 4% |  | 262 | 45% |  | 15 | 3% |  | 28 | 5% |  | 39 | 7% |  | 66 | 11% |  |
|  | 2 | 346 | 100% | 256 | 74% |  | 31 | 9% |  | 227 | 66% |  | 31 | 9% |  | 41 | 12% |  | 44 | 13% |  | 52 | 15% |  |
|  | 3+ | 230 | 100% | 200 | 87% |  | 56 | 24% |  | 167 | 73% |  | 37 | 16% |  | 62 | 27% |  | 89 | 39% |  | 111 | 48% |  |
|  |  |  |  |  |  |  |  |  |  |  |  |  |  |  |  |  |  |  |  |  |  |  |  |  |
| IV | 1 | 1128 | 100% | 130 | 12% | <0.0001 | 31 | 3% | 0.01 | 898 | 80% | <0.0001 | 6 | 1% | 0.005 | 11 | 1% | <0.0001 | 19 | 2% | <0.0001 | 33 | 3% | <0.0001 |
|  | 2 | 801 | 100% | 589 | 74% | 0.31 | 63 | 8% | 0.02 | 716 | 89% | <0.0001 | 59 | 7% | 0.36 | 29 | 4% | <0.0001 | 61 | 8% | 0.003 | 76 | 9% | <0.0001 |
|  | 3+ | 388 | 100% | 325 | 84% | 0.39 | 80 | 21% | 0.39 | 359 | 93% | <0.0001 | 66 | 17% | 0.07 | 92 | 24% | 0.72 | 145 | 37% | 0.75 | 164 | 42% | 0.01 |
|  |  |  |  |  |  |  |  |  |  |  |  |  |  |  |  |  |  |  |  |  |  |  |  |  |

| **Supplemental Table 3.** Multivariable logistic regression model for odds to develop metastases in patients diagnosed with colon cancer (N=31,285). The model adjusts for sex, age at diagnosis, anatomical site, and histological type. | | | | | | | | | | | | | | | | | | | | | | | | | | | | | | | |  |
| --- | --- | --- | --- | --- | --- | --- | --- | --- | --- | --- | --- | --- | --- | --- | --- | --- | --- | --- | --- | --- | --- | --- | --- | --- | --- | --- | --- | --- | --- | --- | --- | --- |
| ***Patient characteristics*** | **Any metastasis** | | | **Thorax** | | | | **Peritoneum** | | | | **Liver** | | | | **Other Gastro-intestinal** | | | | **Nervous system** | | | | **Bone** | | | | **Other** | | | | **Total with metastases** |
|  | **OR** | **95 % CI** | | **%*** | **OR** | **95 % CI** | | **%** | **OR** | **95 % CI** | | **%** | **OR** | **95 % CI** | | **%** | **OR** | **95 % CI** | | **%** | **OR** | **95 % CI** | | **%** | **OR** | **95 % CI** | | **%** | **OR** | **95 % CI** | |  |
| All |  |  |  | 32% |  |  |  | 21% |  |  |  | 70% |  |  |  | 10% |  |  |  | 5% |  |  |  | 8% |  |  |  | 18% |  |  |  | 30% |
| Sex |  |  |  |  |  |  |  |  |  |  |  |  |  |  |  |  |  |  |  |  |  |  |  |  |  |  |  |  |  |  |  |  |
| *Men* | 1 |  |  | 33% | 1 |  |  | 20% | 1 |  |  | 73% | 1 |  |  | 9% | 1 |  |  | 4% | 1 |  |  | 9% | 1 |  |  | 16% | 1 |  |  | 32% |
| *Women* | 0.9 | 0.8 | 0.9 | 31% | 0.9 | 0.8 | 1.0 | 23% | 1.1 | 1.0 | 1.2 | 67% | 0.8 | 0.8 | 0.9 | 10% | 1.0 | 0.9 | 1.1 | 5% | 1.1 | 0.9 | 1.4 | 7% | 0.7 | 0.6 | 0.8 | 21% | **1.2** | 1.1 | 1.3 | 28% |
| Age at diagnosis |  |  |  |  |  |  |  |  |  |  |  |  |  |  |  |  |  |  |  |  |  |  |  |  |  |  |  |  |  |  |  |  |
| *<60* | 1 |  |  | 33% | 1 |  |  | 30% | 1 |  |  | 71% | 1 |  |  | 10% | 1 |  |  | 5% | 1 |  |  | 10% | 1 |  |  | 20% | 1 |  |  | 38% |
| *60-69* | 0.9 | 0.8 | 0.9 | 33% | 0.9 | 0.8 | 1.0 | 21% | 0.6 | 0.5 | 0.7 | 73% | 0.9 | 0.8 | 1.0 | 10% | 0.9 | 0.7 | 1.1 | 5% | 0.9 | 0.7 | 1.1 | 8% | 0.7 | 0.6 | 0.9 | 20% | 0.9 | 0.8 | 1.1 | 34% |
| *70-79* | 0.7 | 0.6 | 0.7 | 33% | 0.8 | 0.7 | 0.9 | 19% | 0.4 | 0.4 | 0.5 | 71% | 0.7 | 0.6 | 0.8 | 9% | 0.7 | 0.6 | 0.8 | 4% | 0.6 | 0.5 | 0.8 | 7% | 0.5 | 0.4 | 0.7 | 17% | 0.6 | 0.5 | 0.7 | 29% |
| *>79* | 0.5 | 0.5 | 0.5 | 28% | 0.5 | 0.4 | 0.6 | 18% | 0.3 | 0.3 | 0.4 | 65% | 0.5 | 0.4 | 0.5 | 10% | 0.6 | 0.5 | 0.7 | 4% | 0.4 | 0.3 | 0.6 | 6% | 0.4 | 0.3 | 0.5 | 17% | 0.5 | 0.4 | 0.6 | 23% |
| Anatomical site |  |  |  |  |  |  |  |  |  |  |  |  |  |  |  |  |  |  |  |  |  |  |  |  |  |  |  |  |  |  |  |  |
| *Proximal colon* | 1 |  |  | 30% | 1 |  |  | 23% | 1 |  |  | 69% | 1 |  |  | 11% | 1 |  |  | 5% | 1 |  |  | 7% | 1 |  |  | 18% | 1 |  |  | 29% |
| *Distal colon* | **1.1** | 1.0 | 1.1 | 35% | **1.2** | 1.1 | 1.3 | 19% | 0.9 | 0.8 | 0.9 | 71% | **1.1** | 1.0 | 1.1 | 8% | 0.8 | 0.7 | 0.9 | 4% | 0.8 | 0.7 | 1.0 | 9% | **1.2** | 1.1 | 1.4 | 18% | 1.1 | 1.0 | 1.2 | 32% |
| Histology |  |  |  |  |  |  |  |  |  |  |  |  |  |  |  |  |  |  |  |  |  |  |  |  |  |  |  |  |  |  |  |  |
| *Adenocarcinoma* | 1 |  |  | 33% | 1 |  |  | 19% | 1 |  |  | 73% | 1 |  |  | 10% | 1 |  |  | 5% | 1 |  |  | 8% | 1 |  |  | 18% | 1 |  |  | 30% |
| *Signet ring/Mucinous AD* | 0.9 | 0.8 | 1.0 | 26% | 0.7 | 0.6 | 0.9 | 43% | **2.2** | 1.9 | 2.5 | 48% | 0.6 | 0.5 | 0.6 | 11% | 1.0 | 0.8 | 1.3 | 3% | 0.6 | 0.4 | 1.0 | 6% | 0.8 | 0.6 | 1.0 | 23% | **1.2** | 1.0 | 1.4 | 28% |
| Bold values indicate significantly higher odds, and underlined values indicate significantly lower odds. | | | | | | | | | | | | | | | | | | | | | | | | | | | | | | | | |
| *Percentages indicate the frequency of specified metastases in relation to patients with any metastasis. E.g. "of all proximal colon cancer patients with metastases, 69 % have liver metastases." | | | | | | | | | | | | | | | | | | | | | | | | | | | | | | | | |

| **Supplemental Table 4.** Multivariable logistic regression model for odds to develop metastases in patients diagnosed with rectal cancer (N=17,811). The model adjusts for sex, age at diagnosis, and histological type. | | | | | | | | | | | | | | | | | | | | | | | | | | | | | | | | | | |  |
| --- | --- | --- | --- | --- | --- | --- | --- | --- | --- | --- | --- | --- | --- | --- | --- | --- | --- | --- | --- | --- | --- | --- | --- | --- | --- | --- | --- | --- | --- | --- | --- | --- | --- | --- | --- |
| ***Patient characteristics*** | **Any metastasis** | | | **Thorax** | | | | **Peritoneum** | | | | **Liver** | | | | **Other Gastro-intestinal** | | | | **Nervous system** | | | | | **Bone** | | | | | | **Other** | | | | **Total with metastases** |
|  | **OR** | **95 % CI** | | **%*** | **OR** | **95 % CI** | | **%** | **OR** | **95 % CI** | | **%** | **OR** | **95 % CI** | | **%** | **OR** | **95 % CI** | | **%** | | **OR** | **95 % CI** | | **%** | | **OR** | | **95 % CI** | | **%** | **OR** | **95 % CI** | |  |
| All (OR rectum vs. colon) | 1.0 | 1.0 | 1.1 | 47% | **1.5** | 1.5 | 1.6 | 8% | 0.4 | 0.3 | 0.4 | 70% | 1.0 | 0.9 | 1.0 | 7% | 0.7 | 0.6 | 0.8 | | 8% | **1.7** | 1.5 | 2.0 | | 12% | | **1.5** | 1.4 | 1.7 | 15% | 0.9 | 0.9 | 0.9 | 31% |
| Sex |  |  |  |  |  |  |  |  |  |  |  |  |  |  |  |  |  |  |  | |  |  |  |  | |  | |  |  |  |  |  |  |  |  |
| *Men* | 1 |  |  | 46% | 1 |  |  | 8% | 1 |  |  | 72% | 1 |  |  | 7% | 1 |  |  | | 7% | 1 |  |  | | 13% | | 1 |  |  | 13% | 1 |  |  | 32% |
| *Women* | 0.9 | 0.9 | 1.0 | 49% | 1.0 | 0.9 | 1.1 | 8% | 1.0 | 0.8 | 1.2 | 65% | 0.8 | 0.8 | 0.9 | 6% | 0.8 | 0.7 | 1.0 | | 9% | 1.2 | 1.0 | 1.5 | | 12% | | 0.9 | 0.8 | 1.1 | 18% | **1.3** | 1.1 | 1.4 | 30% |
| Age at diagnosis |  |  |  |  |  |  |  |  |  |  |  |  |  |  |  |  |  |  |  | |  |  |  |  | |  | |  |  |  |  |  |  |  |  |
| *<60* | 1 |  |  | 47% | 1 |  |  | 11% | 1 |  |  | 73% | 1 |  |  | 8% | 1 |  |  | | 10% | 1 |  |  | | 15% | | 1 |  |  | 16% | 1 |  |  | 38% |
| *60-69* | 0.9 | 0.8 | 0.9 | 49% | 0.9 | 0.8 | 1.0 | 8% | 0.6 | 0.5 | 0.8 | 73% | 0.9 | 0.8 | 0.9 | 6% | 0.7 | 0.5 | 0.9 | | 9% | 0.8 | 0.6 | 1.0 | | 13% | | 0.8 | 0.7 | 1.0 | 15% | 0.8 | 0.7 | 1.0 | 34% |
| *70-79* | 0.7 | 0.7 | 0.8 | 49% | 0.8 | 0.7 | 0.9 | 8% | 0.6 | 0.4 | 0.7 | 66% | 0.6 | 0.6 | 0.7 | 8% | 0.8 | 0.6 | 1.0 | | 8% | 0.6 | 0.5 | 0.8 | | 12% | | 0.6 | 0.5 | 0.8 | 14% | 0.7 | 0.6 | 0.8 | 30% |
| *>79* | 0.5 | 0.5 | 0.6 | 41% | 0.5 | 0.4 | 0.6 | 7% | 0.4 | 0.3 | 0.5 | 64% | 0.5 | 0.4 | 0.5 | 6% | 0.5 | 0.3 | 0.6 | | 4% | 0.3 | 0.2 | 0.4 | | 8% | | 0.3 | 0.2 | 0.4 | 16% | 0.6 | 0.5 | 0.7 | 24% |
| Histology |  |  |  |  |  |  |  |  |  |  |  |  |  |  |  |  |  |  |  | |  |  |  |  | |  | |  |  |  |  |  |  |  |  |
| *Adenocarcinoma* | 1 |  |  | 47% | 1 |  |  | 8% | 1 |  |  | 70% | 1 |  |  | 7% | 1 |  |  | | 8% | 1 |  |  | | 12% | | 1 |  |  | 12% | 1 |  |  | 31% |
| *Signet ring/Mucinous AD* | 1.0 | 0.8 | 1.1 | 40% | 0.8 | 0.6 | 1.0 | 16% | **2.0** | 1.4 | 2.9 | 54% | 0.7 | 0.6 | 0.8 | 7% | 1.1 | 0.7 | 1.8 | | 3% | 0.3 | 0.0 | 0.7 | | 16% | | 1.3 | 0.9 | 1.8 | 16% | **1.9** | 1.5 | 2.5 | 31% |
| Bold values indicate significantly higher odds, and underlined values indicate significantly lower odds. | | | | | | | | | | | | | | | | | | | | | | | | | | | | | | | | | | | |
| *Percentages indicate the frequency of specified metastases in relation to patients with any metastasis. E.g. "of all rectal cancer patients with metastases, 70 % have liver metastases." | | | | | | | | | | | | | | | | | | | | | | | | | | | | | | | | | | | |
